# The Fallacies of the Breast MRI: A Case Study

**DOI:** 10.7759/cureus.39898

**Published:** 2023-06-03

**Authors:** Ekta Mishra, Narinder Kaur, Uma Handa, Gursimran Singh Anand

**Affiliations:** 1 Department of Radiodiagnosis, Government Medical College and Hospital, Chandigarh, Chandigarh, IND; 2 Department of Pathology, Government Medical College and Hospital, Chandigarh, Chandigarh, IND

**Keywords:** benign and malignant breast lesions, diagnostic mammography, mammography, palpable lump, mr mammography

## Abstract

Magnetic resonance imaging (MRI) of breasts using diffusion-weighted imaging and dynamic contrast enhancement is now well-established imaging for the evaluation and characterization of suspicious breast lesions, where it has become a problem-solving tool. Breast lesions are characterized according to their morphological features and enhancement characteristics. Breast MRI is helpful in the evaluation of breast lesions in patients with dense breasts and women with breast implants and to differentiate scars and recurrence. However, this technique has its own limitations, a few of which are elucidated in the present case report.

## Introduction

Breast magnetic resonance imaging (MRI) has the capability of providing three-dimensional spatial information and better visual differentiation of breast lesions from normal breast tissue based on differences in the vascularity and permeability of the masses [1]. Compared to conventional breast imaging, breast MRI is functional imaging.

Two major imaging characteristics of a breast mass used to interpret findings on breast MRI include the morphological appearance of the mass and its enhancement pattern. Morphological assessment includes size, shape, margins, internal features, and diffusion restriction. Enhancement characteristics are assessed by evaluating the changes in signal intensity (SI) of the lesion on pre-contrast and multiple post-contrast images on dynamic contrast-enhanced MRI (DCEMRI). Irregular shape, non-circumscribed margins, diffusion restriction, and heterogenous/rim enhancement with washout kinetic curve are suggestive of a malignant mass. An oval or round shape, circumscribed margins, and homogenous enhancement with a persistent kinetic curve on DCEMRI are the findings suggestive of a benign breast mass.

Many studies have been carried out in the literature to demonstrate the role of breast MRI in the assessment of suspicious breast masses. In a study conducted by Fischer et al. (1999), it was concluded that the breast contrast-enhanced MRI was highly sensitive to invasive breast cancer. MRI might reveal unsuspected multifocal, multicentric, or contralateral breast carcinoma and result in subsequent treatment changes [2].

Nonetheless, some studies have also shown low specificity for breast MRI in the discernment of benign and malignant lesions of the breast [3-5].

## Case presentation

A 72-year-old female presented with clinical complaints of pain and a lump in her left breast for the past one day with no history of fever. On physical examination, a tender, small nodule was seen in the peri-areolar region of the left outer quadrant of the breast. The overlying skin was normal. She had a family history of invasive breast cancer in her maternal aunt. She underwent conventional mammography, breast ultrasound/sonomammography, and breast MRI, followed by fine needle aspiration cytology (FNAC).

**Figure 1:**
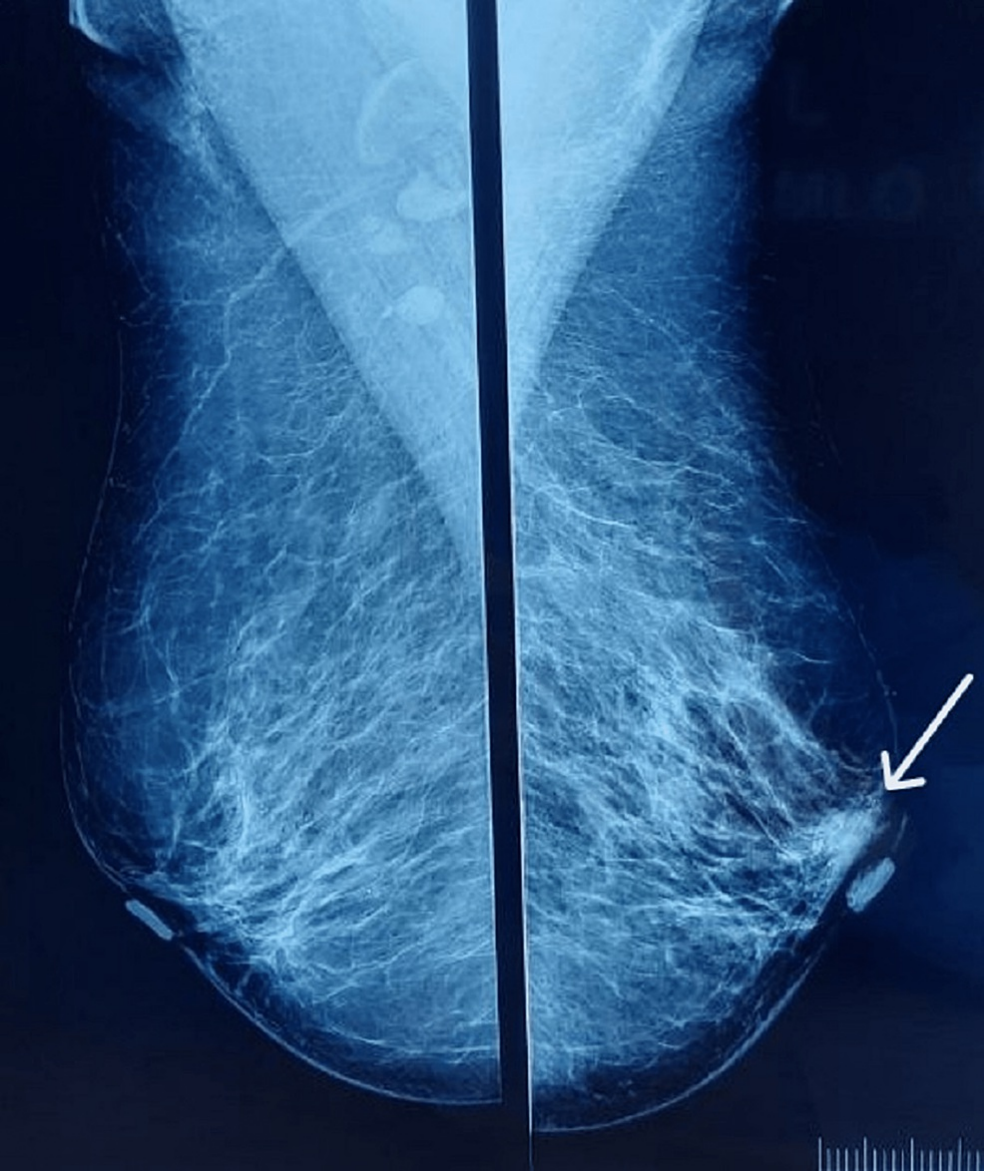
Conventional mammography: mediolateral oblique (MLO) view MLO: mediolateral oblique

**Figure 2:**
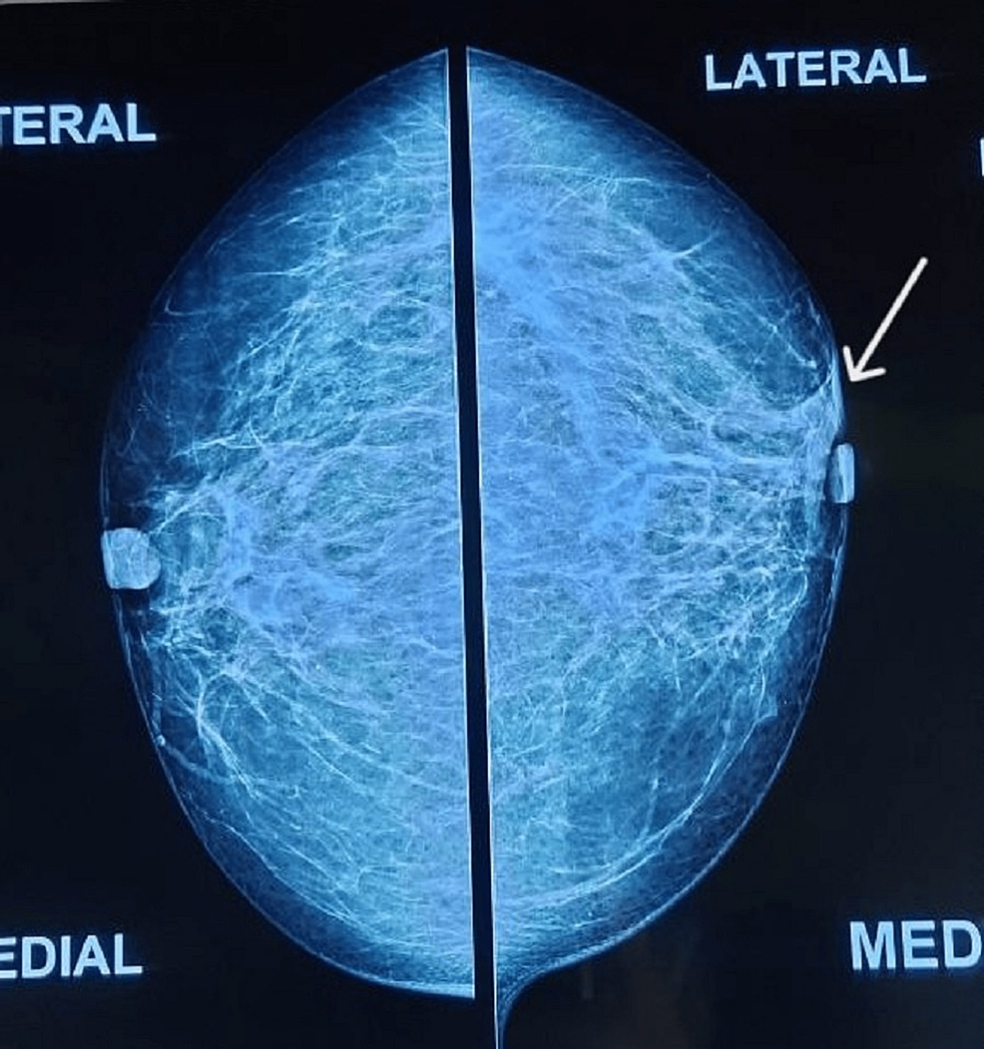
Conventional mammography: craniocaudal (CC) view Conventional mammography revealed bilateral dense breasts for age, and an irregular, partially obscured, equal-density mass lying parallel to the skin is seen in the upper outer quadrant of the left breast in the peri-areolar region (marked by an arrow). CC: craniocaudal

Conventional mammography revealed bilateral dense breasts for age and an irregular, partially obscured, equal-density mass lying parallel to the skin that measures 8x5 mm in the upper outer quadrant of the left breast, in the peri-areolar region. No associated architectural distortion was seen. The overlying skin appears normal. A few lymph nodes were seen in the right axilla with a maintained shape and fatty hila, measuring up to 10 mm in maximum dimension (Figure 1 and Figure 2).

On sonomammography correlation, an irregular, heterogeneously hypoechoic mass was seen, which was parallel to the skin and measured 1x0.6 cm in size at the three o'clock position in the peri-areolar region. It shows minimal internal vascularity. Underlying a few ducts appeared prominent, with a maximum caliber of up to 2.6 mm; however, no intraductal contents were seen. A few lymph nodes with preserved fatty hilum and normal shape were seen in the right axilla, with a maximum size of 0.5x0.8 mm. The breast mass was categorized as breast imaging reporting and data system (BI-RADS) 4 on conventional mammography and sonomammography. The patient subsequently underwent a breast MRI, which also included DCEMRI for the characterization of the breast mass.

**Figure 3:**
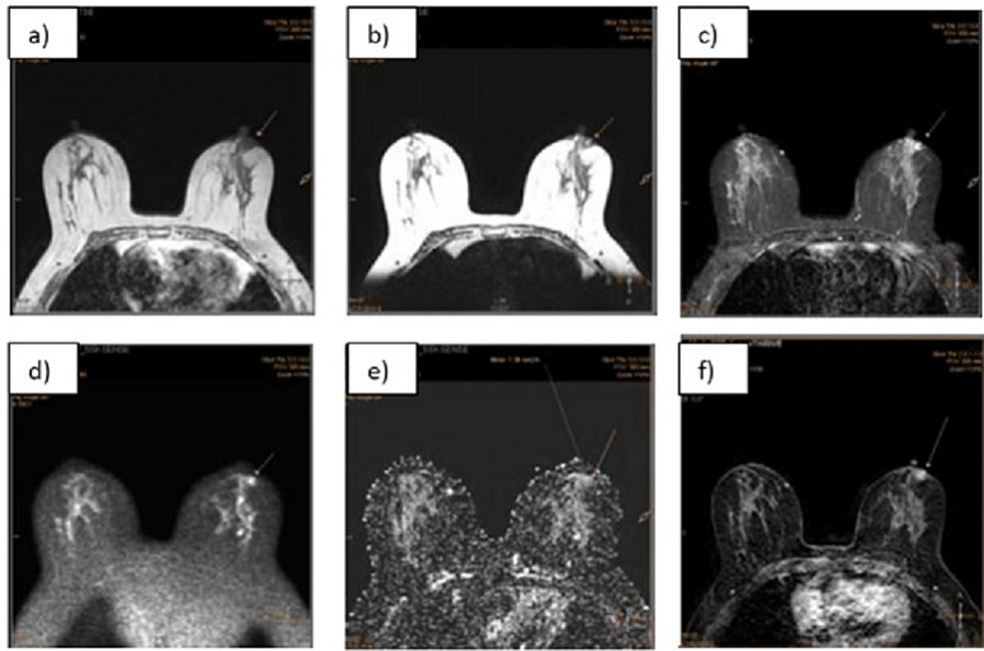
MR mammography: morphological features a) An irregular non-circumscribed mass (marked with an arrow) is showing a hypointense signal on T1WI; b) The lesion shows a hyperintense signal on T2WI; c) The irregular mass shows a hyperintense signal on STIR images in the peri-areolar region of the left breast; (d,e) Patchy diffusion restriction is seen in the lesion on DWI/ADC images (marked with arrow); (f) Heterogeneous avid contrast enhancement is seen in the lesion on post-contrast images STIR: short-tau inversion recovery; DWI: diffusion-weighted imaging; ADC: apparent diffusion coefficient

**Figure 4:**
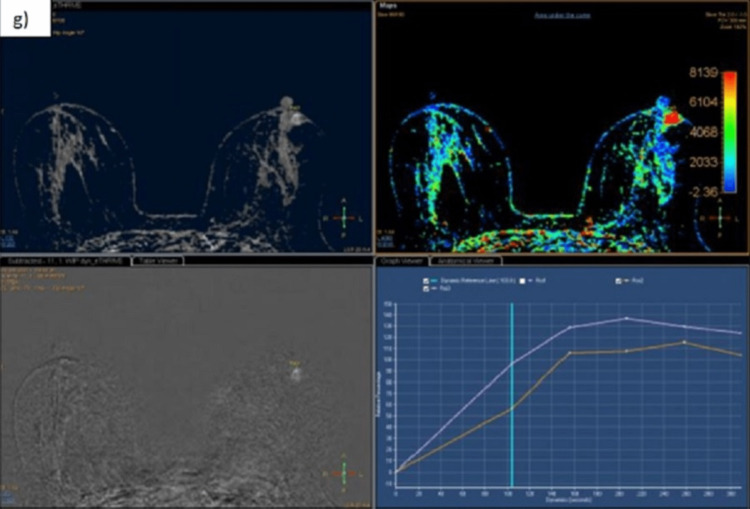
MR mammography: dynamic evaluation On post-contrast images, heterogenous enhancement and Fast/type 3 kinetic curve were seen (g). MR: magnetic resonance

There is evidence of an irregular non-circumscribed lesion with irregular micro-lobulated margins showing a hypointense signal on T1 weighted image (WI) and a heterogeneously hyperintense signal on T2/short-tau inversion recovery (STIR) images, measuring 7.8x6.8 mm (APxTR) in size at the three o'clock position in the peri-areolar region of the left breast. Patchy diffusion restriction is noted with ADC values up to 1.32x10-3 mm2/sec. On dynamic post-contrast images, it shows heterogenous enhancement. On the initial enhancement phase, it shows a rapid curve, and on the delayed phase, a washout/type 3 kinetic curve is seen (Figure 3 and Figure 4).

The right breast showed no abnormal enhancement of focus, mass, or non-mass enhancement. The mass was neither upgraded nor downgraded and was kept as BI-RADS 4.

**Figure 5:**
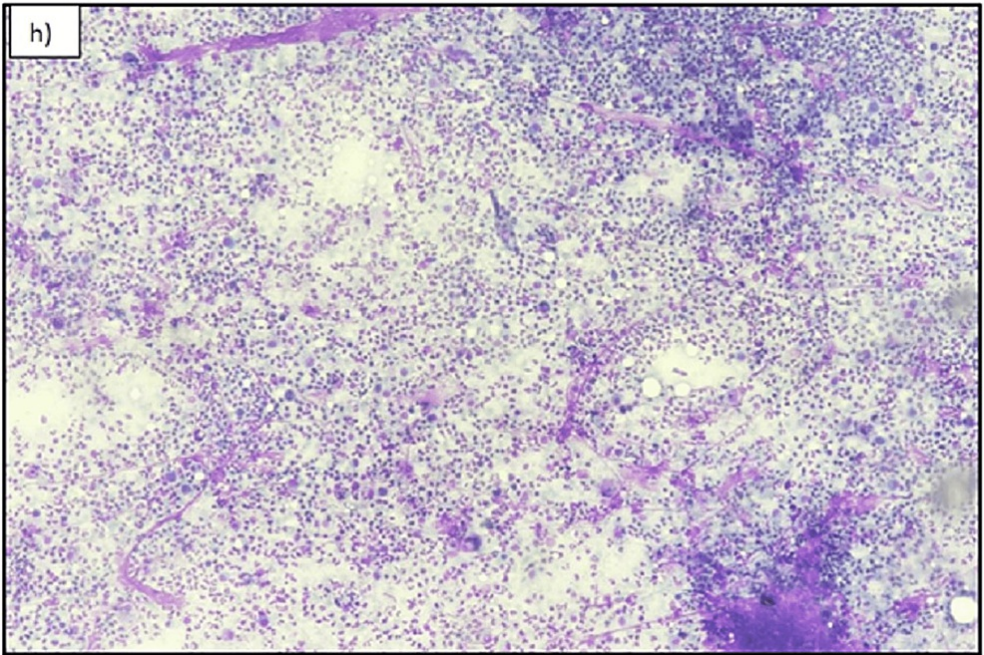
Fine needle aspiration cytology (FNAC) smear Photomicrograph of May-Grunwald-Giemsa (MGG) slide shows h) (X200) and i) (X400) Abscess. Cytological smears show dense mixed inflammation composed of neutrophils, lymphocytes, plasma cells, and histiocytes. FNAC: fine needle aspiration cytology; MGG: May-Grunwald-Giemsa

**Figure 6:**
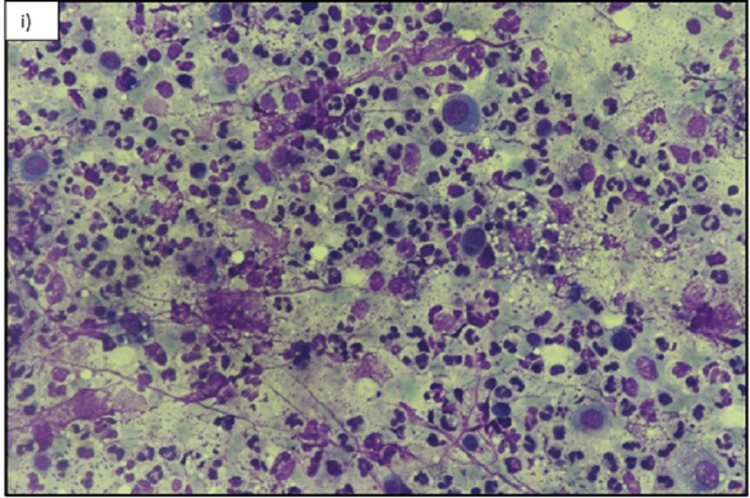
Fine needle aspiration cytology (FNAC) cytological smear Photomicrograph shows May-Grunwald-Giemsa (MGG) h) (X200) and i) (X400) Abscess. Cytological smears show dense mixed inflammation composed of neutrophils, lymphocytes, plasma cells, and histiocytes. FNAC: fine needle aspiration cytology; MGG: May-Grunwald-Giemsa

The patient subsequently underwent FNAC of this mass under ultrasound guidance. The final pathology report showed an extensive inflammatory infiltrate comprising neutrophils, lymphocytes, and macrophages with phagocytosis. Extensive capillary proliferation, nuclear debris, and benign ductal epithelial cells were also seen (Figure 5 and Figure 6). The Ziehl-Neelsen stain for acid-fast bacilli (AFB) was negative. The findings were consistent with an abscess.

## Discussion

Breast masses are primarily evaluated on MRI by their morphological characteristics (shape, size, and margin), internal features such as diffusion restriction, and enhancement characteristics (internal enhancement pattern, shape of time-intensity curves). In multiple studies, it has been emphasized that morphological characteristics supersede enhancement characteristics in determining the nature of a lesion [6].

Certainly, breast MRI is more sensitive than conventional imaging for detecting multifocal or multicentric disease; however, there is evidence in the literature that few females have undergone more extensive surgery or invasive interventions as a result of breast MRI without clear evidence of benefit. There is no role for breast MRI as a substitute for conventional imaging (mammography) or for screening women at average risk of breast cancer. It also does not play a role in routine diagnostic testing for women with symptoms [7].

Breast MRI has been associated with higher rates of breast surgeries and mastectomies [8-10] and treatment delays of 22.4 days, as reported in the literature by Bleicher et al. in 2008.

## Conclusions

The breast mass in the present case report had an irregular shape and margin. On post-contrast images, it showed heterogenous enhancement and demonstrated curves with rapid initial enhancement and a washout curve on the delayed phase. All these features pointed towards a more sinister diagnosis and malignant pathology. However, on pathological examination, it turned out to be an abscess.

Though MRI of the breast is highly sensitive for detecting breast malignancy, it needs to be interpreted with caution due to the high rate of false positives. Our reference case summarizes the fallacies of both the morphological and enhancement patterns of DCEMRI. Hence, we recommend that a breast mass suspected of malignancy on an MRI be subjected to a biopsy, or FNAC, before surgery.
